# Decrease of AIM2 mediated by luteolin contributes to non-small cell lung cancer treatment

**DOI:** 10.1038/s41419-019-1447-y

**Published:** 2019-03-04

**Authors:** Qian Yu, Minda Zhang, Qidi Ying, Xin Xie, Shuwen Yue, Bending Tong, Qing Wei, Zhaoshi Bai, Lingman Ma

**Affiliations:** 1 State Key Laboratory of Natural Medicines, School of Life Science and Technology, China Pharmaceutical University, Nanjing, 210009 Jiangsu China; 2 State Key Laboratory of Natural Medicines, Department of Physiology, China Pharmaceutical University, Nanjing, 210009 Jiangsu China; 3 Department of Pharmacology, School of Pharmacy, China Pharmaceutical University, Nanjing, 210009 Jiangsu China; 4 Jiangsu Cancer Hospital & Jiangsu Institute of Cancer Research & The Affiliated Cancer Hospital of Nanjing Medical University, Nanjing, 210009 Jiangsu China

## Abstract

Non-small cell lung cancer (NSCLC) is one of the most common malignancies in the world. Although extensive studies showed that luteolin exhibited antitumor effects against NSCLC, the mechanism has not been fully established. In the present study, we found that luteolin significantly reduced the expression of absent in melanoma 2 (AIM2) at both mRNA and protein levels leading to the suppression of AIM2 inflammasome activation, which induced G2/M phase arrest and inhibited epithelial–mesenchymal transition (EMT) in NSCLC. Furthermore, the inhibitory effects of luteolin on NSCLC cells were abolished by the knockdown of AIM2. On the contrary, the antitumor effects of luteolin could be notably reversed by the overexpression of AIM2. In addition, luteolin reduced poly(dA:dT)-induced caspase-1 activation and IL-1β cleavage in NSCLC cells. These findings suggested that AIM2 was essential to luteolin-mediated antitumor effects. The antitumor effects of luteolin, which were closely associated with AIM2, were also confirmed in the A549 and H460 xenograft mouse models. Collectively, our study displayed that the antitumor effects of luteolin on NSCLC were AIM2 dependent and the downregulation of AIM2 might be an effective way for NSCLC treatment.

## Background

Non-small cell lung cancer (NSCLC) is the most common type of lung cancer and remains as a serious public health concern^1^. At present, NSCLC is broadly divided into four categories: lung adenocarcinoma, lung squamous cell carcinoma, large cell carcinoma, and undifferentiated NSCLC^2^. Most patients with NSCLC present with locally advanced and metastatic disease at diagnosis. Although some emerging new target drugs or biomedical technique have been verified for NSCLC treatment, chemotherapy has been the mainstay of treatment at present^3,4^. However, chemotherapy has many drawbacks especially for drug resistance and non-selected toxicity^5^.

Absent in melanoma 2 (AIM2), as a receptor for cytosolic dsDNA, combines apoptosis-associated speck-like protein containing a CARD (ASC) adaptor and pro-caspase-1 to form an AIM2 inflammasome^6,7^. This multi-protein complex senses host- and pathogen-associated cytoplasmic DNA and induces caspase-1 activation, resulting in proteolytic cleavage of the proinflammatory cytokines pro-IL-1β and pro-IL-18 to active forms^8–10^. In addition, the interaction of inflammation and cancer is now generally accepted, so it is not strange that AIM2 also plays a vital role in cancers. There are some reports that involved in the correlation between AIM2 expression and cancer progression. For example, AIM2 mRNA levels were significantly upregulated in oral squamous cell carcinoma and Epstein-Barr virus-induced nasopharyngeal carcinoma^11,12^. As previous study reported that the overexpression of AIM2 could promote AIM2 inflammasome formation and activation in hepatocarcinoma cells^13^. AIM2 was highly expressed in NSCLC cell lines^14^. The activated AIM2 inflammasome could promote the maturation of proinflammatory cytokines. Importantly, dysregulation of inflammatory cytokines in the lung is thought to contribute to inflammatory diseases and NSCLC^10^. Moreover, studies showed that the activation of inflammasome also promoted the epithelial–mesenchymal transition (EMT) of tumor cells, which played an important role in the procession of malignant tumor^15^. Therefore, we speculated that the inhibition of AIM2 inflammasome could exhibit antitumor effects in NSCLC. Therefore, the detailed mechanism of AIM2 in NSCLC should be put forward.

**Fig. 1:**
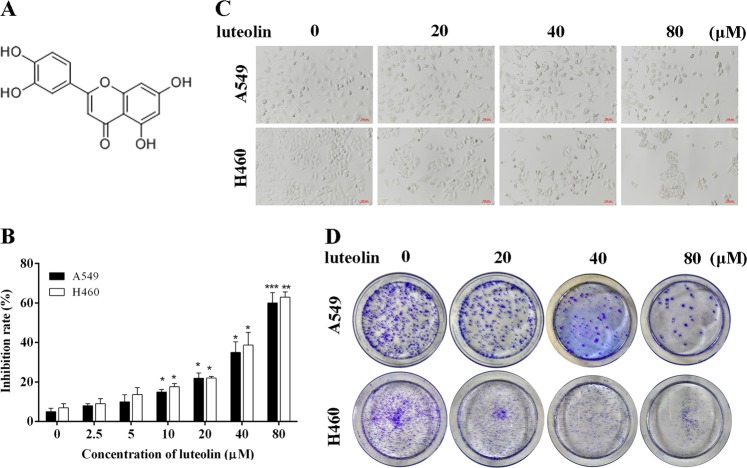
Luteolin reduced the proliferation of A549 and H460 cells. **a** The chemical structure of luteolin. **b** MTT assay for cell growth of A549 and H460 cells after treatment with different concentrations of luteolin. **c** A549 and H460 cells were treated with luteolin at different concentrations for 24 h. Cell morphology was captured by Nikon microscope (scale bar = 100 μm). **d** Representative images of the colony formation assay for A549 and H460 cells. *P *<* 0.05, **P < 0.01, ***P < 0.001 vs control

Luteolin (Fig. 1a), as a natural flavonoid, possesses a wide spectrum of pharmacological actions including anti-hyperlipidemia, anti-tussive and anti-asthmatic, antianaphylaxis, anti-arthritis, as well as anti-inflammation in clinical treatments^16–21^. It was worth noting that the anti-inflammatory activity was the major pharmacological mechanism of luteolin, which involved with regulating various mediators of inflammation and influencing various signaling pathways related to inflammation^22^. Studies confirmed that inflammation played a critical role in all stages, from initiation through progression to deterioration of cancer^23^. Interestingly, most reports also established the inhibitory effects of luteolin on a large range of cancers^24–28^. While some researches have been carried out on luteolin, the mechanism by which the therapeutic effect of luteolin on NSCLC has not been fully established, particularly the molecular connection between luteolin and AIM2 remaining largely elusive. In this study, we indicated that luteolin suppressed the activation of AIM2 inflammasome by the downregulation of AIM2, thereby inducing G2/M phase arrest and inhibiting EMT in A549 and H460 cells. To further verify the roles of AIM2 under luteolin treatment, siAIM2 and AIM2 overexpression plasmid were used. Silencing of AIM2 abolished the inhibitory effects of luteolin on G2/M phase arrest and EMT, whereas AIM2 overexpression displayed effects opposite to those of siAIM2 in luteolin-regulated cell cycle and EMT. The in vivo study reproduced our findings in vitro, luteolin possessed strong antitumor effects on A549 and H460 xenograft animals. We concluded that the downregulation of AIM2 is an effective therapeutic strategy mediated by luteolin, which is associated with how luteolin exerts its antitumor effects in NSCLC.

## Results

### Luteolin suppressed the proliferation of NSCLC cells

To determine the effect of luteolin on NSCLC cell growth, A549, H460, and H226 cells were treated with various concentrations of luteolin and cell inhibition rates were assessed by 3-(4,5-Dimethyl-2-thizolyl)-2,5-diphenyltertazolium bromide (MTT) assay. As shown in Fig. 1b, c, luteolin showed significantly inhibitory activity on A549 and H460 cell, and IC_50_ values of luteolin at 24 h were determined as 66.51 and 59.10 μM, respectively. Meanwhile, normal human bronchial epithelial cells 16HBE was tested to further confirm whether luteolin was non-toxic to normal lung cells at a dosage of growth inhibiting on tumor cells. Importantly, 16HBE cells were not significantly sensitized to luteolin within the range of experimental concentrations, and the inhibition rate of 16HBE was under 10% after treatment with luteolin for 24 or 48 h (Fig. S1A). We also observed that luteolin concentration dependently inhibited cell growth of H226 cells with IC_50_ value of 81.71 μM at 48 h (Fig. S1B). In addition, luteolin also inhibited cell growth of A549/Taxol cells (Fig. S1C). In addition, the morphological observations found that luteolin-treated A549, H460, and H226 cells showed cell shrinkage and extensive cell detachment from the substratum (Fig. 1c and Fig. S1D).

In order to confirm these results, we investigated whether luteolin treatment had an impact on the clonogenic activity of A549, H460, and H226 cells. In contrast with the control group, luteolin concentration dependently inhibited the colony numbers of A549, H460, and H226 cells (Fig. 1d and Fig. S1E). All these data suggested that luteolin suppressed proliferation of NSCLC cells in vitro.

### Luteolin-induced G2/M phase arrest in A549 and H460 cells

**Fig. 2:**
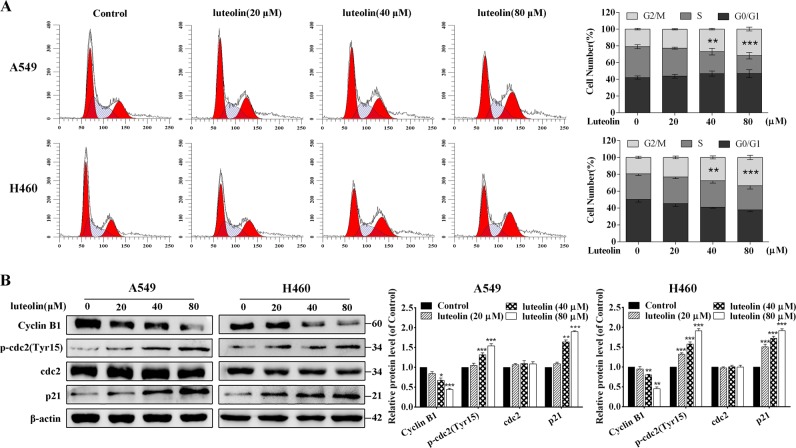
Luteolin-induced G2/M cell cycle arrest in A549 and H460 cells. **a** Representative flow histograms depicted cell cycle distribution and summarized flow cytometry data. **b** Western blot analysis for cyclin B1, p-cdc2 (Tyr15), cdc2 and p21 in A549 and H460 cells treated with luteolin at different concentrations for 24 h. *P < 0.05, **P < 0.01, ***P < 0.001 vs control

It is well known that uncontrolled cell proliferation caused by dysregulation of the cell cycle progression is the hallmark of cancer^29^. To further investigate the mechanism of luteolin-mediated growth inhibition of A549 and H460 cells, cell cycle distribution was examined by flow cytometry. We found that luteolin treatment resulted in the accumulation of cells at G2/M phase in A549 and H460 cells (Fig. 2a).

The cyclin B1/cdc2 complex, known as the M phase-promoting factor, functions in the mitosis-promoting factor in G2/M phase, and the suppression of cyclin B1 can cause the accumulation of cells at G2/M phase^30^. Meanwhile, p21, a universal cyclin-dependent kinases (CDKs) inhibitor, plays a key role in cell cycle^31^. Therefore, we determined the expression of cell cycle-related proteins by western blot in order to investigate the molecular basis of G2/M phase arrest. As shown in Fig. 2b, luteolin significantly decreased the expression of cyclin B1, but enhanced the expression of p21 in both A549 and H460 cells. Meanwhile, no change was noted in total cdc2, but significant upregulation of p-cdc2 (Tyr15), which negatively regulates the activation of the cyclin B1/cdc2 complex was observed. Altogether, these results strongly indicated that luteolin blocked the G2/M transition of A549 and H460 cells through the inhibition of cyclin B1/cdc2 activity and the increase of p21 expression.

### Luteolin inhibited EMT in A549 and H460 cells

**Fig. 3:**
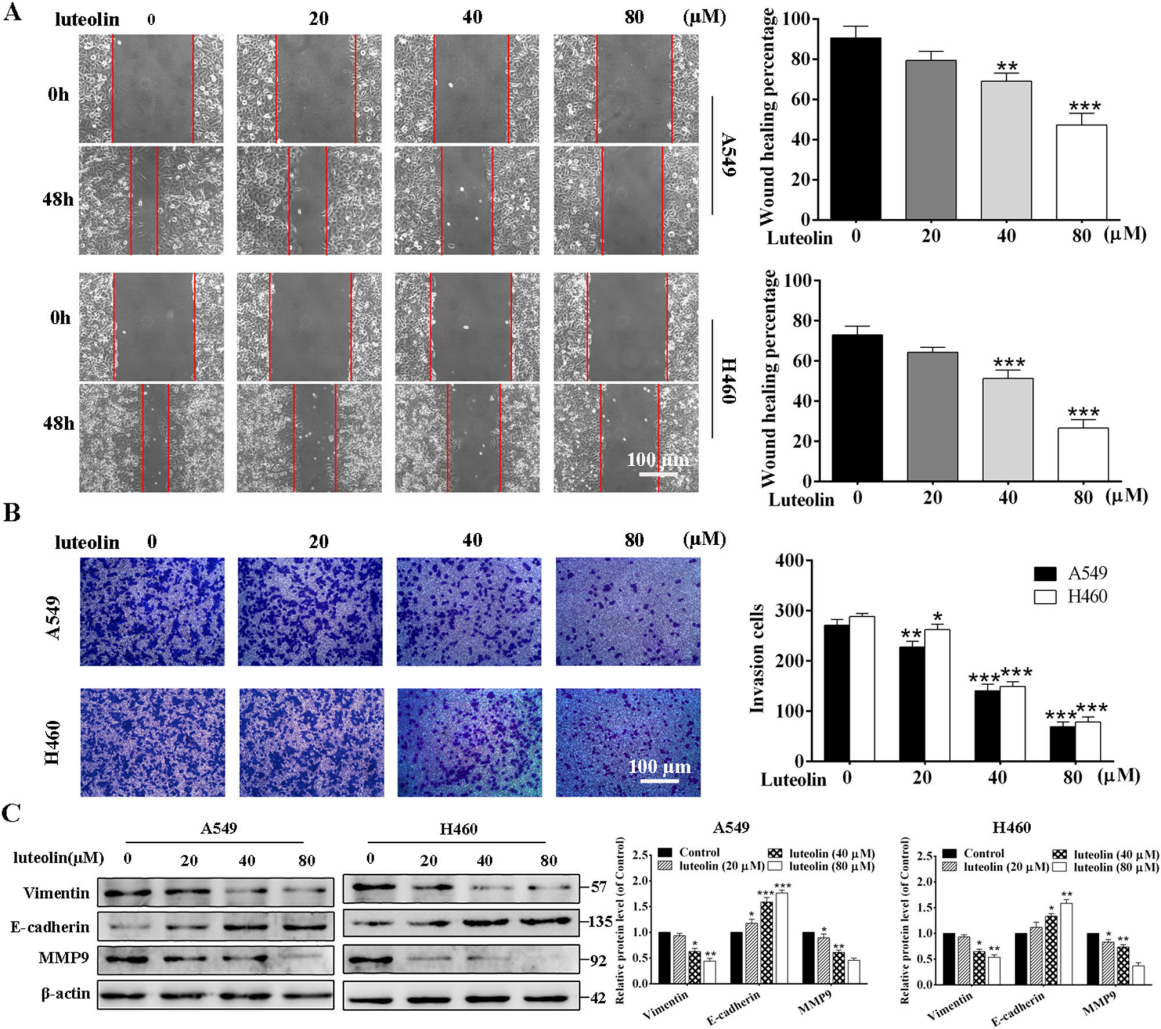
Luteolin decreased the invasive and migratory abilities of A549 and H460 cells. The migration (**a**) and the invasion (**b**) of A549 and H460 cells with the treatment of luteolin (0, 20, 40, and 80 μM) were examined by scratch wound-healing assay and transwell assay, respectively (scale bar = 100 μm). **c** Western blot assay for protein expression of Vimentin, E-cadherin, and MMP9 in A549 and H460 cells treated with luteolin (0, 20, 40, and 80 μM) for 24 h. *P < 0.05, **P < 0.01, ***P < 0.001 vs control

EMT is known as a characteristic of malignancy, which reflected on enhancing the invasion and metastasis abilities of tumor cells^32^. Therefore, we next explored the changes of migratory and invasive abilities of A549 and H460 cells under luteolin treatment by wound-healing assay and invasion assay. The wound-healing assay showed that the migratory ability of A549 cells suppressed by luteolin and the wounded area of luteolin (80 μM) group was still large after culturing for 48 h (Fig. 3a). Consistently, invasion assay demonstrated that luteolin concentration dependently decreased the invasiveness of A549 cells. Even the lowest concentration of luteolin (20 μM) showed partial inhibition of cell invasion (Fig. 3b). Similar results were observed in H460 cells (Fig. 3b).

To further determine the underlying molecular basis of these alterations, we detected the characteristic EMT markers including E-cadherin, Vimentin, and matrix metalloproteinase 9 (MMP9) by western blot. In both A549 and H460 cells, the expressions of Vimentin and MMP9 were significantly decreased, but E-cadherin was significantly increased in a concentration-dependent manner after luteolin treatment (Fig. 3c). Taken together, these results suggested that luteolin could greatly suppress EMT by decreasing the expressions of Vimentin and MMP9 and increasing the expression of E-cadherin in A549 and H460 cells.

### The activation of AIM2 inflammasome was suppressed by luteolin in A549 and H460 cells

**Fig. 4:**
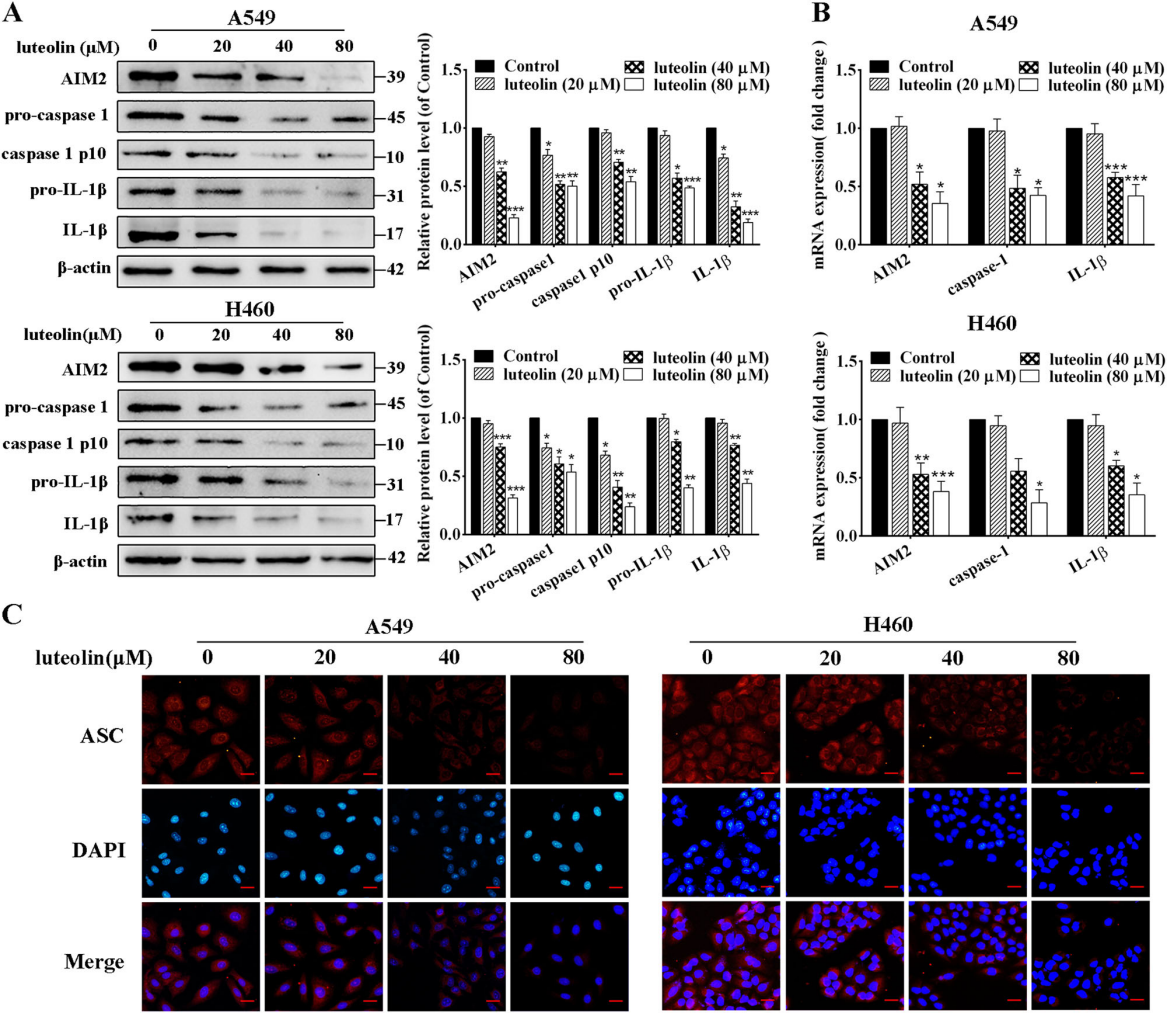
Luteolin inhibited the activation of AIM2 inflammasome in vitro. **a** Western blot analysis for the protein expressions of AIM2, pro-caspase-1, caspase-1 p10, pro-IL-1β, and IL-1β in A549 and H460 cells. **b** RT-qPCR detection for the mRNA expressions of AIM2, caspase-1, and IL-1β. **c** Immunofluorescence analysis of endogenous ASC in cells treated by luteolin for 24 h (scale bar = 25 μm). *P < 0.05, **P < 0.01, ***P < 0.001 vs control

Previous studies showed that dysregulation of chronic inflammation was responsible for many types of cancers, and proinflammatory cytokines secreted by inflammasome were involved in this process. AIM2 dysregulation in the lung is thought to contribute to inflammatory diseases and NSCLC^33^. We found that the expression of AIM2 was highly expressed in H226, A549, A549/Taxol, and H460 cells compared with 16HBE cells (Fig. S2A). In addition, luteolin concentration dependently decreased the expressions of AIM2, pro-caspase-1, caspase-1 p10, pro-IL-1β, and IL-1β in A549, H460, and H226 cells (Fig. 4a and Fig. S2B). Consistent with this, Real-time quantitative polymerase chain reaction (RT-qPCR) assay displayed a similar trend with decreased mRNA levels of AIM2, caspase-1 and IL-1β (Fig. 4b and Fig. S2C). Importantly, formation of ASC speck was considered as another hallmark of inflammasome activation^34^. The inhibitory effect of luteolin on AIM2 inflammasome activation was also proved by ASC immunostaining in A549 and H460 cells (Fig. 4c).

**Fig. 5:**
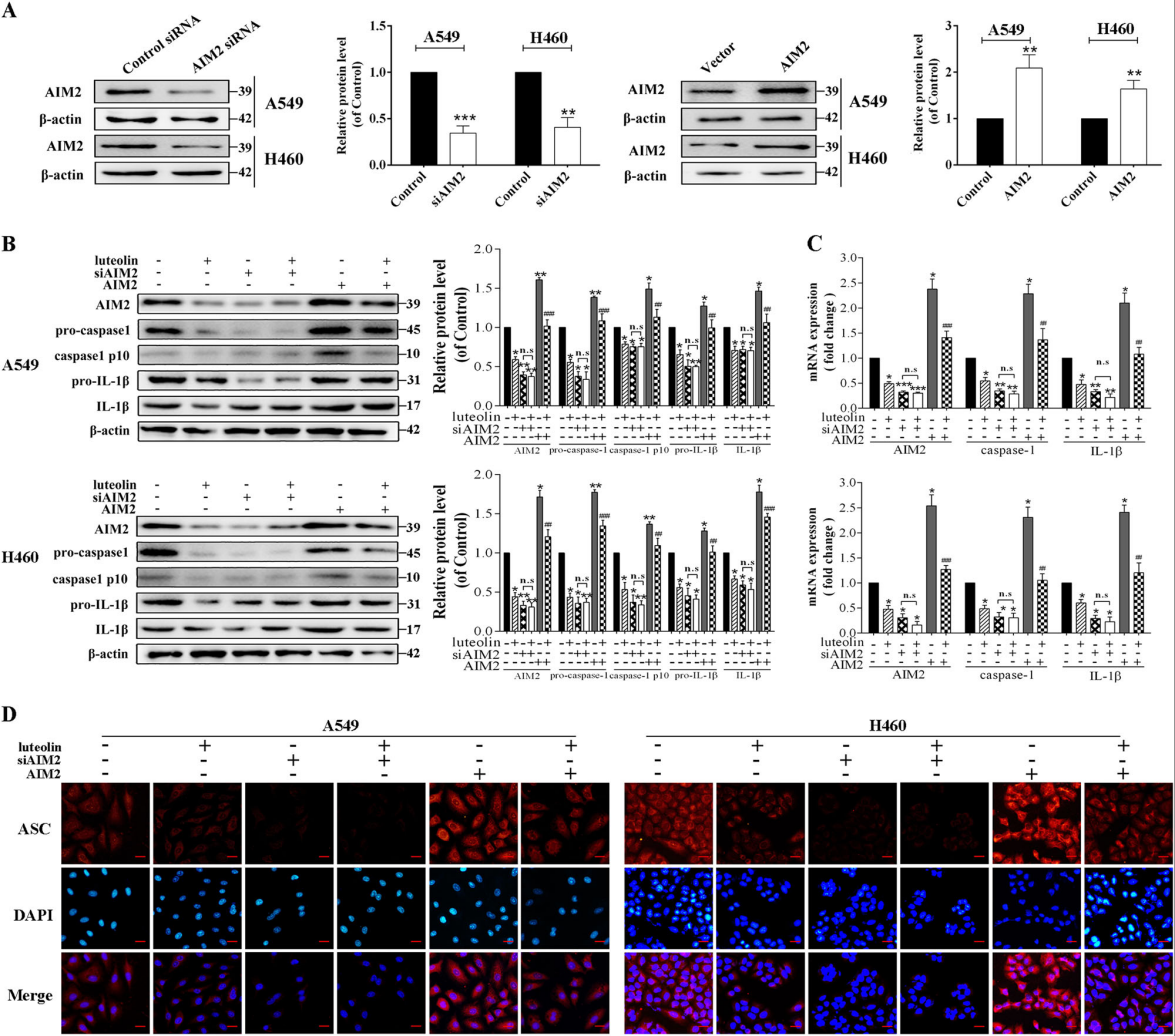
Luteolin suppressed AIM2 inflammasome through downregulating the expression of AIM2 in A549 and H460 cells. **a** Western blot analysis of AIM2 expression in A549 and H460 cells transfected with siAIM2 or AIM2 overexpression plasmid. Cells were transfected with siAIM2 or AIM2 overexpression plasmid, with or without the treatment of luteolin (40 μM) for 24 h. **b** The expressions of AIM2, pro-caspase-1, caspase-1 p10, pro-IL-1β and IL-1β were determined by western blot. **c** RT-qPCR were performed to detect the mRNA levels of AIM2, caspase-1 and IL-1β. **d** Cells were pretreated with siAIM2 or AIM2 overexpression plasmid before treating with or without luteolin for 24 h to detect the localization of ASC by immunofluorescence (scale bar = 25 μm). *P < 0.05, **P < 0.01, ***P < 0.001 vs control; ^#^*P* < 0.05, ^##^*P* < 0.01, ^###^*P* < 0.001 vs luteolin (40 μM). NS no significant difference

In order to further verify the role of AIM2 under luteolin treatment, AIM2 siRNA and AIM2 overexpression plasmid were used. Successful transfections were demonstrated by western blot (Fig. 5a and Fig. S3A). Luteolin-mediated caspase-1 activation and IL-1β maturation as reflected in the downregulation of caspase-1 p10 and IL-1β were nearly completely abolished when AIM2 was absent in NSCLC cells (Fig. 5b and Fig. S3B). By contrast, AIM2 overexpression notably reversed the inhibitory effects of luteolin on caspase-1 activation and pro-IL-1β maturation in NSCLC cells (Fig. 5b and Fig. S3B). RT-qPCR assay displayed an obvious decrease in mRNA levels of AIM2, caspase-1 and IL-1β after the addition of luteolin (Fig. 5c and Fig. S3C). Immunofluorescence assay showed that AIM2 overexpression induced ASC speck formation, whereas AIM2 silencing significantly reduced ASC speck formation. Luteolin also reduced ASC speck formation in AIM2 overexpression plasmid-pretreated cells (Fig. 5d and Fig. S3D).

The poly(dA:dT), a synthetic analog of microbial dsDNA, is a inducer of AIM2 inflammasome^8^. Prior study also indicated that AIM2 plays an important role in AIM2 inflammasome activation by poly(dA:dT)^5^. Consistent with previous studies, we also found that poly(dA:dT) stimulation resulted in AIM2 inflammasome activation. In addition, luteolin reduced poly(dA:dT)-induced caspase-1 activation and IL-1β cleavage in A549, H460 and H226 cells (Fig. S4). Taken together, these results demonstrated that luteolin inhibited the activation of AIM2 inflammasome *via* downregulating the expression of AIM2.

### The antitumor effects of luteolin in A549 and H460 cells were correlated with AIM2

**Fig. 6:**
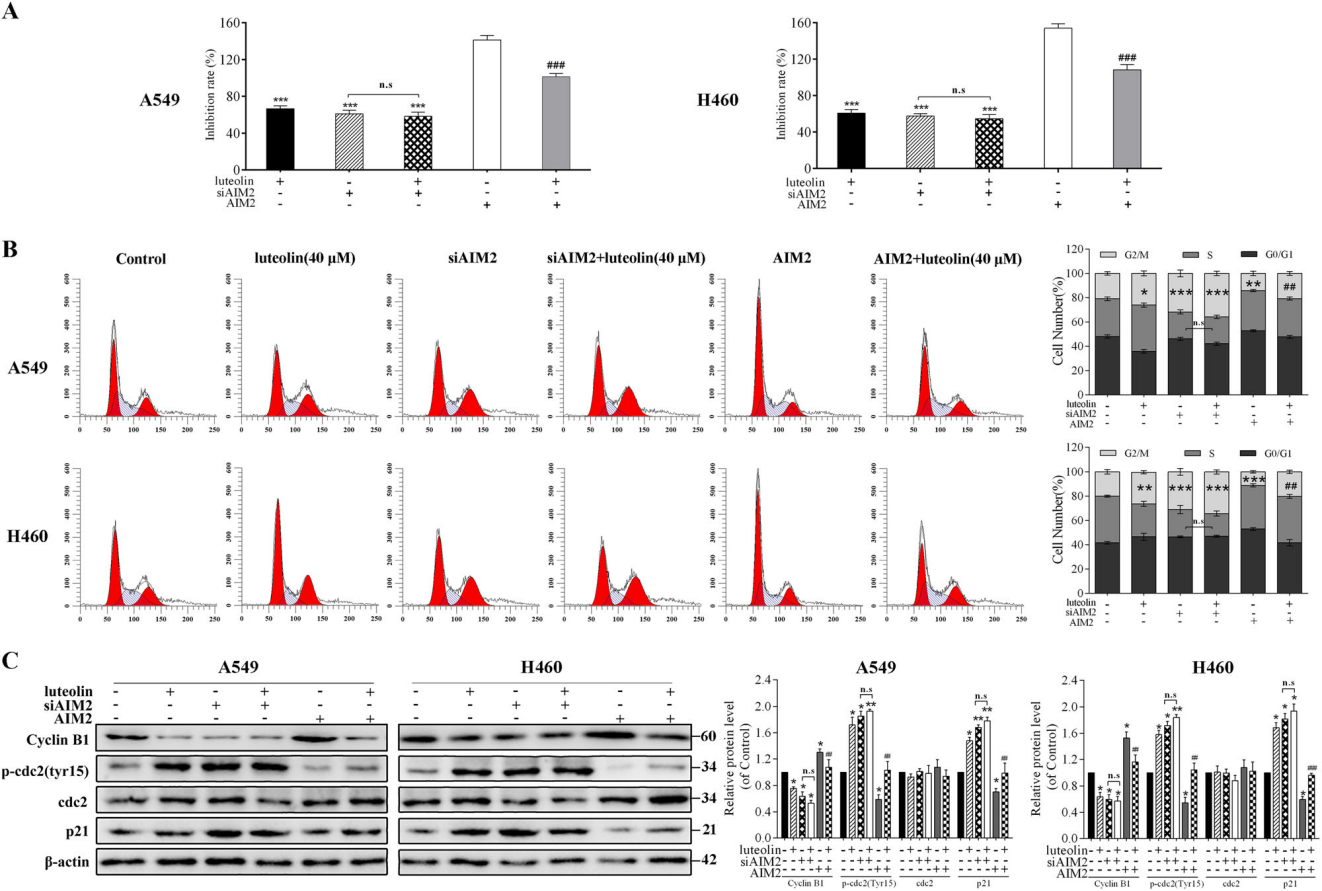
Luteolin reduced cell proliferation and causes G2/M phase arrest in A549 and H460 cells. Cells were pretreated with siAIM2 or AIM2 overexpression plasmid before treating with or without luteolin (40 μM) for 24 h. **a** MTT method for cell proliferation analysis. **b** Representative flow histograms depicted cell cycle distribution and summarized flow cytometry data. **c** Western blot for the expressions of cyclin B1, p-cdc2 (Tyr15), cdc2 and p21. *P < 0.05, **P < 0.01, ***P < 0.001 vs control, ^#^*P* < 0.05, ^##^*P* < 0.01, ^###^*P* < 0.001 vs luteolin (40 μM). NS no significant difference

To verify whether the AIM2 mediated by luteolin was involved in cell proliferation, MTT assay was used. The results showed that the anti-proliferative effect of luteolin in NSCLC cells was impaired when AIM2 was absent (Fig. 6a). By contrast, AIM2 overexpression notably rescued the anti-proliferative effect of luteolin (Fig. 6a). We further detected the influence of AIM2 in cell cycle distribution. As shown in Fig. 6b, siAIM2 significantly induced G2/M phase arrest in both cell lines, but the proportion of cells in G2/M phase could not be further increased by the addition of luteolin in siAIM2-pretreated cells. Similarly, the expressions of p-cdc2 (Tyr15), p21, and cyclin B1 exhibited no significant differences between siAIM2 alone treatment group and the combined treatment of siAIM2 and luteolin group (Fig. 6b, c). Moreover, luteolin-induced G2/M phase arrest in A549 and H460 cells was significantly rescued through upregulating the expressions of p-cdc2 (Tyr15), p21, and cyclin B1 by AIM2 overexpression plasmid (Fig. 6b, c).

**Fig. 7:**
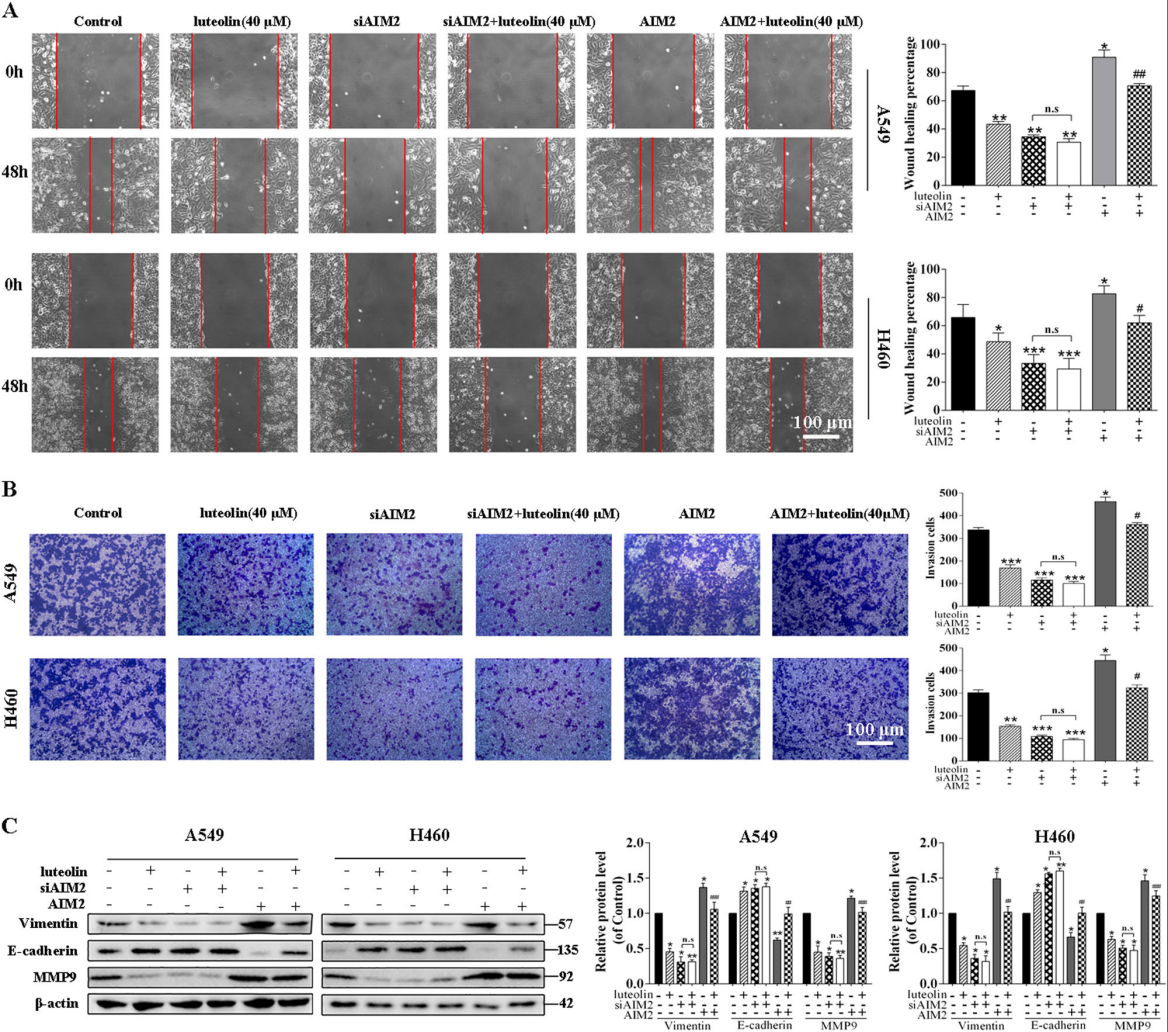
Luteolin decreased the migratory and invasive capabilities by downregulating AIM2 expression in A549 and H460 cells. Cells transfected with siAIM2 or AIM2 overexpression plasmid were treated with or without luteolin (40 μM) for 24 h. The migration (**a**) and the invasion (**b**) of A549 and H460 cells were examined by scratch wound-healing assay and transwell assay, respectively (scale bar = 100 μm). **c** The expressions of Vimentin, E-cadherin, and MMP9 were determined by western blot. *P < 0.05, **P < 0.01, ***P < 0.001 vs control, ^#^*P* < 0.05, ^##^*P* < 0.01, ^###^*P* < 0.001 vs luteolin (40 μM). NS no significant difference

Next, we investigated the mechanisms underlying the inhibitory effects of luteolin on cell migration and invasion. As shown in Fig. 7a, b, siAIM2 significantly inhibited A549 and H460 cells migration and invasion. Meanwhile, the changes of EMT-related markers were obviously influenced by siAIM2; while in siAIM2-pretreated groups, no significant differences of Vimentin, E-cadherin, and MMP9 expressions were observed upon luteolin treatment (Fig. 7c). On the contrary, AIM2 overexpression reversed the inhibitory effects of luteolin on cell migration and invasion (Fig. 7a, b). In addition, we found that the upregulation of E-cadherin and the downregulation of Vimentin and MMP9 after luteolin treatment were also reversed by AIM2 overexpression plasmid (Fig. 7c). Data above collectively suggested that the antitumor effects of luteolin in NSCLC cells were at least in part, if not all, AIM2 dependent.

### Luteolin inhibited the growth of A549 and H460 xenografts in vivo

**Fig. 8:**
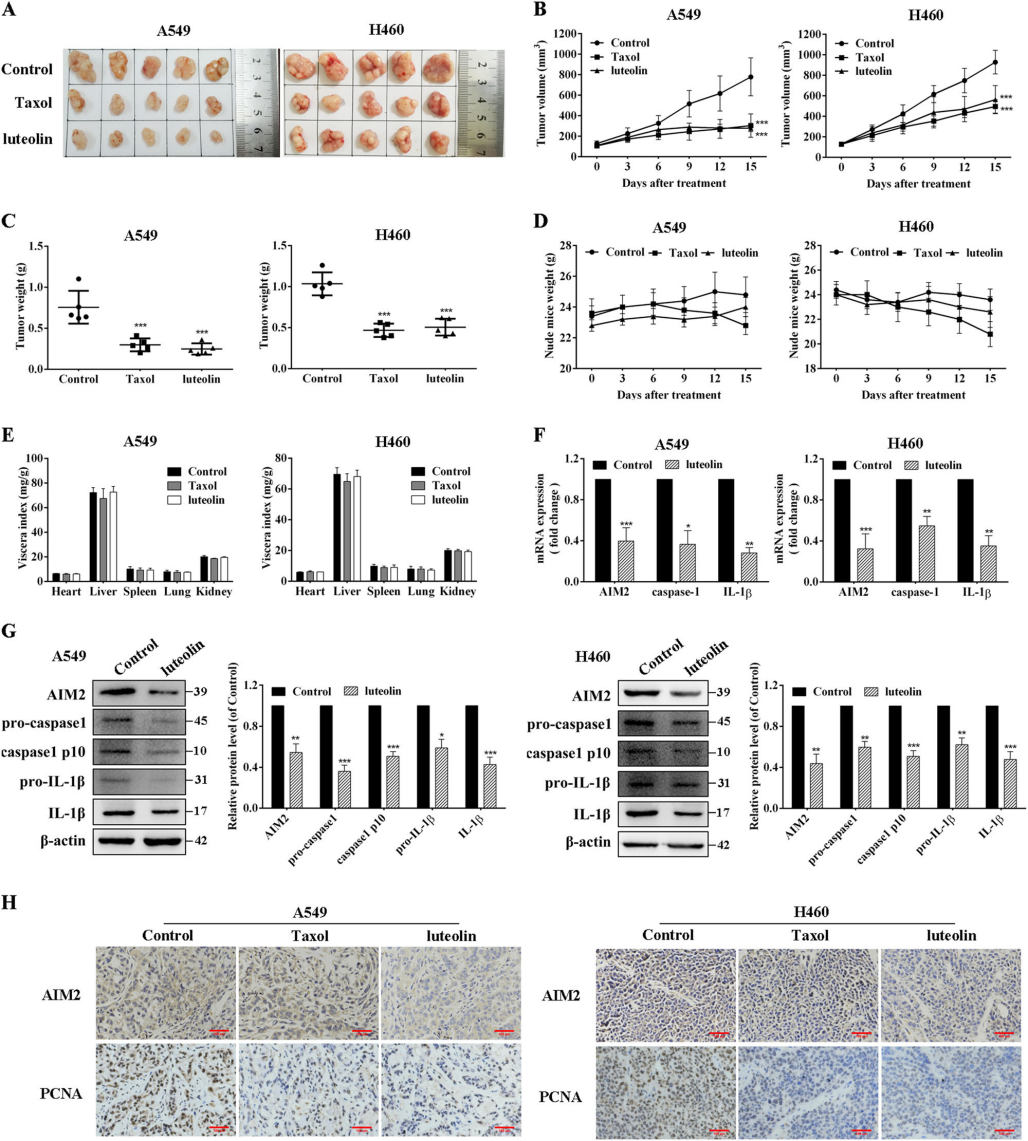
Luteolin inhibited NSCLC growth in vivo. **a** Images of resected xenograft samples. **b** Average tumor volumes. **c** Average tumor weights. **d** The body weights of mice. **e** The viscera indexes of mice main organs. **f** RT-qPCR assay for the expressions of AIM2, caspase-1, and IL-1β in tumor tissues. **g**, **h** Western blot analysis for the expressions of AIM2, pro-caspase-1, caspase-1 p10, pro-IL-1β, and IL-1β in tumor tissues. **i**, **j** Immunohistochemical staining of PCNA and AIM2 in tumor tissues (scale bar = 100 μm). *P < 0.05, **P < 0.01, ***P < 0.001 vs control

In this study, we further validated the antitumor effects of luteolin in vivo by using nude mouse xenograft models. Both A549 and H460 cell-bearing mice showed a significant growth inhibition of the tumors with administration of luteolin and taxol, compared with model groups (Fig. 8a). The average volume and weight of tumors were dramatically decreased in luteolin and taxol groups (Fig. 8b, c). As shown in Fig. 8d, e, the body weight and viscera index (the ratio of visceral weight to body weight [mg/g]) of luteolin groups had no significant differences with those of model group in both A549 and H460 cell-bearing mice, suggesting a low toxicity of luteolin in vivo. Differently, an obvious body weight loss was found in the taxol-treated mice (Fig. 8d).

Next, we evaluated the levels of AIM2 in tumor tissues and found that luteolin administration significantly downregulated AIM2, caspase-1, and IL-1β expressions at mRNA levels (Fig. 8f). Similarly, decreased expressions of AIM2, pro-caspase-1, caspase-1, pro-IL-1β, and IL-1β in luteolin-treated groups were further verified by western blot (Fig. 8g). In addition, the results of immunohistochemistry showed that AIM2, as well as the cell proliferation marker Proliferating Cell Nuclear Antigen (PCNA) in tumor tissues were significantly downregulated by luteolin (Fig. 8h). Therefore, our study showed that luteolin inhibited tumor growth of A549 and H460 cells in vivo through AIM2 inhibition.

## Discussion

In this study, we found that AIM2 was essential to luteolin-mediated antitumor effects in NSCLC both in vitro and in vivo. Our results also indicated that AIM2 had an important role in inhibition of proliferation and EMT by luteolin in NSCLC. Similarly, the antitumor effects of luteolin, which were closely associated with AIM2, were also confirmed in the A549 and H460 xenograft mouse models. Therefore, our data proved that the antitumor effects of luteolin in NSCLC cells were at least in part, if not all, AIM2 dependent.

AIM2 functions as a receptor for cytosolic DNA, which contributes to against infection with microbial or viral pathogens and tissue damage^35^. Upon sensing the cytosolic DNA, AIM2 recruits an adaptor protein ASC and pro-caspase-1 to form a functional inflammasome complex, which drives the maturation and secretion of caspase-1 resulting in proteolytic cleavage of the proinflammatory cytokines pro-IL-1β to an active form. In addition, overexpression of AIM2 promoted pulmonary inflammation and correlated with poor prognosis^36^. Currently, accumulative evidences have suggested that luteolin possesses significant anti-inflammatory effects by reducing proinflammatory cytokine production. Since AIM2 plays an important role in inflammation, we investigated the effects of luteolin on AIM2 in NSCLC. Consistent with these studies, our study showed that the expression of AIM2 was highly expressed in H226, A549, A549/Taxol, and H460 cells compared with 16HBE cells. Moreover, luteolin alleviated inflammation in A549, H460, and H226 cells by influencing the expression of AIM2 and decreasing proinflammatory cytokines production. Poly(dA:dT), a synthetic analog of microbial dsDNA, has been reported to stimulate AIM2 inflammasome activation^5^. In this study, we found that luteolin could reduce poly(dA:dT)-induced caspase-1 activation and IL-1β maturation in A549, H460, and H226 cells. In addition, knockdown of AIM2 inhibited the migration and invasion of A549 and H460 cells as compared with control cells, whereas AIM2 overexpression promoted these activities in both cell lines. Therefore, several lines of evidence have demonstrated that AIM2 not only acted an important role in inflammatory responses, but also played critical roles in tumorigenesis and metastasis. Importantly, downregulation of AIM2 may be a new effective mechanism for NSCLC treatment by luteolin.

Previous studies showed that dysregulation of chronic inflammation was contributed to about 25% of all human cancers^23^. Links between chronic inflammation and cancer were closely related and interacted with each other, and lung cancer was no exception. On the one hand, chronic inflammation has many tumor-promoting effects, which directly or indirectly results in inflammatory angiogenesis, proliferation, progression, and metastasis^37^. On the other hand, cancer can also lead to inflammation^38^. Both the human cases and experimental animal models found that inflammatory mediators exist in the tumor microenvironment from the early-stage of lung cancer^37^. Interestingly, Nonsteroidal anti-inflammatory drugs (e.g., aspirin and ibuprofen) could reduce the clinical signs and symptoms of inflammation-related conditions, especially for cancers^39,40^. As luteolin significantly relieved inflammation by downregulating the expression of AIM2, we hypothesized that luteolin might exhibit antitumor effects by alleviating inflammatory conditions.

Our data showed that luteolin-mediated inhibition of proliferation was associated with the accumulation of cells at G2/M phase, which was demonstrated by cell cycle-related markers. Cdc2, a subunit of the cyclin B1/cdc2, showed significant increase of phosphorylation at Tyr15 in A549 and H460 cells. The functional complex formed by cdc2 and cyclin B1 plays an indispensible role on the transition from G2 to M phase of cell cycle. Meanwhile, the upregulation of p21, a universal CDK inhibitor, further verified the role of luteolin in G2/M arrest. Thus, the inhibition of G2/M transition induced by luteolin might depend on the inhibition of cyclin B1/cdc2 activity and the increase of p21 expression. Furthermore, EMT is regarded as one of pivotal steps in the progression of lung cancer toward invasion and metastasis^41^. Here, luteolin suppressed the EMT in A549 and H460 cells, which was demonstrated by the upregulation of the epithelial marker E-cadherin and the downregulation of the mesenchymal marker Vimentin and the matrix proteolytic enzyme MMP9. In the present study, luteolin-induced G2/M phase arrest and suppressed the EMT in A549 and H460 cells. Subsequently, siAIM2 and AIM2 overexpression plasmid were used to verify the roles of AIM2 under luteolin treatment. Our data revealed that the regulatory effects of luteolin on the G2/M phase transition were abolished by the addition of siAIM2 or were rescued by the overexpression of AIM2. Similarly, AIM2 was demonstrated to influence the G2 to M transition of the cell cycle in colon cancer^42^. Studies also proved that inflammation is responsible for a pro-invasive and pro-metastatic milieu within tumors^43^. Thus, we investigated the effects of luteolin on EMT in A549 and H460 cells, which were pretreated with siAIM2 or AIM2 overexpression plasmid. In this study, the negative regulations of luteolin on EMT in A549 and H460 cells were nearly completely abolished when AIM2 was absent. The abilities of luteolin to reduce Vimentin and MMP9 expressions and to enhance E-cadherin expression were impaired in AIM2-knockdown A549 and H460 cells. Furthermore, AIM2 overexpression notably reversed the inhibitory effects of luteolin on EMT. These findings agreed with previous studies that AIM2 was associated with the regulation of migration and invasion of cancer cells^42^. Collectively, luteolin significantly reduced AIM2 expression to inhibit the activation of AIM2 inflammasome, resulting in G2/M phase arrest and EMT suppression. Silencing of AIM2 attenuated the antitumor effects of luteolin, whereas the upregulation of AIM2 reversed the antitumor effects of luteolin, indicating that AIM2 might serve as a potential target for luteolin treatment.

Luteolin also exerted potent anticancer activity against A549 and H460 cells in vivo. Compared with taxol, luteolin displayed similar antitumor effects as reflected in the decreasing tumor weights and tumor volumes. In addition, luteolin did not affect the mice body weights and viscera indexes. These results implied that luteolin had a better safety profile and exhibited potent antitumor effects. The in vivo study also reproduced our findings in vitro, that is, luteolin suppressed the expressions of AIM2, pro-caspase-1, caspase-1 p10, pro-IL-1β, and IL-1β in tumor samples from nude mice. Moreover, AIM2 was significantly downregulated in the luteolin-treated mice.

In summary, we identified the capacity of luteolin in inhibition of NSCLC and the possible mechanisms underlying. AIM2 could be identified as a potential target for NSCLC treatment. Luteolin-induced G2/M phase arrest and inhibited EMT via the downregulation of AIM2 in NSCLC cells, indicating that AIM2 might be a novel and promising therapeutic target for NSCLC and proposing a potential molecular mechanism of luteolin treatment.

## Materials and methods

### Chemicals and antibodies

Luteolin ( > 99% pure) was purchased from DASF Bio-Tech Ltd (Nanjing, P.R.C.). Paclitaxel (Taxol) injection was purchased from Cisen Pharmaceutical Co., Ltd (Shandong, P.R.C.). Primary antibodies for AIM2, cyclin B1, cdc2, and phospho-cdc2 (Tyr15), PCNA, β-actin, Vimentin, E-cadherin, MMP9, p21, and Tetramethylrhodamine (TRITC)-conjugated secondary antibodies were gained from Biogot Biotechnology Co., Ltd (Louis Park, Minnesota, USA). Antibodies against ASC, pro-caspase-1, caspase-1 p10, pro-IL-1β, and IL-1β were all gained from Santa Cruz Biotechnology Inc. (Delaware Ave Santa Cruz, CA, USA). Crystal violet, 4′,6-diamidino-2-phenylindole (DAPI) and Lipo6000™ transfection reagent were purchased from Beyotime Biotechnology (Nanjing, P.R.C.). AIM2 overexpression plasmid and AIM2 siRNA were purchased from Shanghai Sango Biotechnology (Shanghai, P.R.C.). Cell cycle detection kit was obtained from KeyGen Biotechnology Co., Ltd (Nanjing, P.R.C.). SYBR Green Master Mix was obtained from Vazyme Biotech Co., Ltd (Nanjing, P.R.C.). Matrigel was purchased from Becton, Dickinson and Company (NY, USA). Poly(dA:dT) was purchased from InvivoGen (CA, USA).

### Cell lines and culture

Cell lines A549, H460, H226, and 16HBE were obtained from Shanghai Cell Bank of Chinese Academy of Sciences. A549/Taxol cells were given by Professor Wu from Shenyang Pharmaceutical University. A549 and H460 cells were cultured with Dulbecco’s modified Eagle's medium supplemented with 10% fetal bovine serum (FBS). 16HBE, H226, and A549/Taxol were maintained in RPMI-1640 medium supplemented with 10% FBS. Both cell lines were grown under 95% humidified air with 5% CO_2_ at 37 °C.

### MTT assay

The routine MTT assay was performed to measure the cell growth inhibitory rates. Briefly, cells were seeded into 96-well plates at density of 5 × 10^3^ cells/well and treated with 0.1% dimethylsulfoxide (DMSO; used as control group) and various concentrations of luteolin for indicated times, respectively. Cell growth inhibition of 50% (IC_50_) was calculated by SPSS 22.0 software.

### Colony formation assay

Cells pretreated by 0.1% DMSO or various concentrations of luteolin were respectively seeded into 60 mm culture dishes at a density of 1000 cells for 2 weeks. The supernatants were discarded, and cells were washed, fixed in 4% paraformaldehyde, stained with crystal violet, and air dried at room temperature.

### Cell cycle analysis

Cells with 60–70% conflunce were synchronized by serum starvation overnight and treated with various concentrations of luteolin for 24 h. After washing with phosphate-buffered saline (PBS), the harvested cells were fixed in 70% cold ethanol overnight at –4 °C. Next day, the fixed cells were washed with PBS and then stained by cell cycle detection kit for 30 min at room temperature. DNA contents and cell cycle distribution were determined by flow cytometry (BD Accuri™ C6 Flow Cytometer, Becton Dickinson, Franklin Lakes, NJ, USA) and were analyzed by Modifit 5.0 software, respectively.

### Wound-healing assay

Cells were seeded into six-well plates for 24 h adherent culture, scraped with a sterile pipette tip and finally treated with various concentrations of luteolin after removing debris by PBS. The scratch area was observed by microscopy at 0 and 48 h.

### Invasion assay

The upper surface of the transwell inserts (8-μm pore size, Corning, NY, USA) were coated with matrigel before serum-free medium containing 1 × 10^5^ cells were loaded. The lower chamber included 500 μl media containing 10% FBS and various concentrations of luteolin. After 24 h, the invaded cells were fixed with 4% paraformaldehyde and stained with crystal violet. Image J was used to count the number of cells in photographed images.

### Western blot

Cells were harvested and lysed by RIPA lysis buffer. After protein quantification by BCA protein assay, the routine western blot procedures were performed as previously described^27^. Briefly, proteins were separated by sodium dodecyl sulfate–polyacrylamide gel electrophoresis and transferred to polyvinylidene difluoride membranes by standard procedures. After blocking with 5% bovine serum albumin (BSA), the membranes were incubated with the primary antibodies overnight at 4 °C, followed by incubation with horseradish peroxidase (HRP)-conjugated secondary antibodies for 2 h at room temperature. The protein bands were identified by enhanced chemiluminescence kits and assessed by Image J.

### Real-time quantitative PCR

Cell total RNA was extracted with TRIzol reagent, reverse transcribed into single-strand complementary DNA (cDNA) and assayed by real-time qPCR using AceQ qPCR SYBR Green Master Mix. The primers were listed as follows: AIM2: (forward) 5′-TGGCAAAACGTCTTCAGGAGG-3′, (reverse) 5′-AGCTTGACTTAGTGGCTTTGG-3′; Caspase-1: (forward) 5′-TTACAGACAAGGGTGCTGAACAA-3′, (reverse) 5′-TGAGGAGCTGGAAAGGAAGAAAG-3′; IL-1β: (forward) 5′-AGGCTGCTCTGGGATTC-3′, (reverse) 5′-GCCACAACAACTGACGC-3′; GAPDH: (forward) 5′-AAGGTCGGAGTCAACGGATTT-3′ (reverse) 5′-AGATGATGACCCTTTTGGCTC-3′. The data were normalized to GAPDH expression and quantified by the 2^-ΔΔCt^ method.

### Immunofluorescence assay

Cells were washed with PBS, fixed with 4% paraformaldehyde, permeabilized with 0.2% Triton X-100 and then blocked with 5% BSA for 1 h followed by incubation with primary antibodies overnight at 4 °C. After washing with PBS, cells were incubated with anti-rabbit TRITC-conjugated secondary antibody. Images were acquired by a fluorescence microscope (Nikon, Tokyo, Japan) after staining the cell nuclei with DAPI.

### Transfection of AIM2 plasmid, AIM2 siRNA, and poly(dA:dT)

AIM2 overexpression plasmid, AIM2 siRNA and poly(dA:dT), were transformed according to the manufacturer’s instruction of Lipo6000™ transfection reagent. The sequence of AIM2 siRNA was as following: (forward) 5′-CCCGAAGAUCAACACGCUUCA-3′, (reverse) 5′-UGAAGCGUGUUGAUCUUCGGG-3′.

### Mouse xenograft assay

Male nude mice (BALB/c nu/nu) (5 weeks old, 18–22 g) were obtained from Comparative Medicine Centre of Yangzhou University (Yangzhou, P.R.C.) and maintained in pathogen-free conditions. A549 (1 × 10^7^ cells/mice) or H460 (5 × 10^6^ cells/mice) cells were injected subcutaneously into the right front axilla of male nude mice. When the tumor volumes reached 100 mm^3^, mice were randomly allocated into three groups (*N* = 5) and intraperitoneally treated with vehicle (5% ethanol + 30% PEG-400 + 65% saline), taxol (10 mg/kg, taxol injection diluted in saline), or luteolin (50 mg/kg, dissolved in the same vehicle as the model group), respectively. The tumor volumes were measured with a digital calliper every 3 days and calculated by the following formula: volume = (length × width^2^)/2. At the end of the experiment, all tumor-bearing mice were sacrificed for obtaining their tumors and organs (heart, liver, spleen, lung, and kidney). All animal experimental procedures were approved by the Institutional Animal Care and Use Committee of China Pharmaceutical University and Jiangsu Cancer Hospital.

### Immunohistochemistry assay

Paraffin-embedded slides were deparaffinized, rehydrated, blocked, and incubated with primary antibodies for 1 h at 37 °C. Then slides were incubated with a secondary antibody labeled with HRP, stained with Diaminobenzidine substrate and counterstained with hematoxylin. The immunoreactivity was assessed by optical microscope.

### Statistical analysis

The data were presented as mean ± SD and were considered statistically significant if *p* < 0.05. The Student’s two-tailed *t*-test was used to assess significance between two groups. Statistical significance of differences between multiple groups was determined by one-way analysis of variance and Dunnett’s comparison. Data management and analysis were performed using SPSS 22.0 software (SPSS, Chicago, IL, USA).

## Supplementary information

The effects of luteolin in 16HBE, H226 and A549/Taxol cells

Luteolin inhibited the activation of AIM2 inflammasome in H226 cells

Luteolin suppressed AIM2 inflammasome through downregulating the expression of AIM2 in H226 cells

Luteolin reduced poly(dA:dT)-induced caspase-1 activation and IL-1β maturation

Proposed mechanisms illustrating the anti-tumor effects of luteolin on NSCLC cells

Supplemental figure legends
