# Supplementary figures and images for: Decrease of AIM2 mediated by luteolin contributes to non-small cell lung cancer treatment
Source: Cell Death Dis. 2019 Mar 4;10(3):218. doi: 10.1038/s41419-019-1447-y (PMC6399355; doi:10.1038/s41419-019-1447-y)

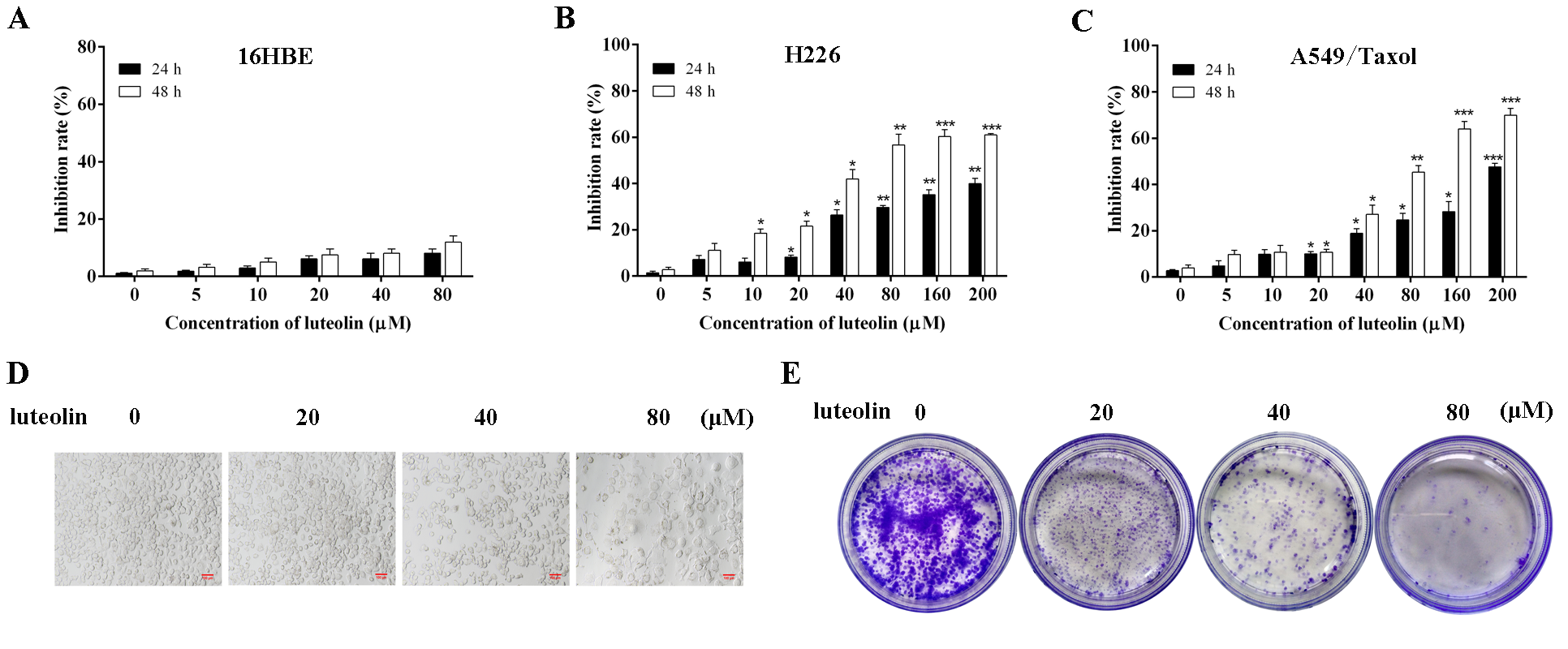

Supplement: Supplementary file 1 — The effects of luteolin in 16HBE, H226 and A549/Taxol cells [file 41419_2019_1447_MOESM1_ESM.tif]

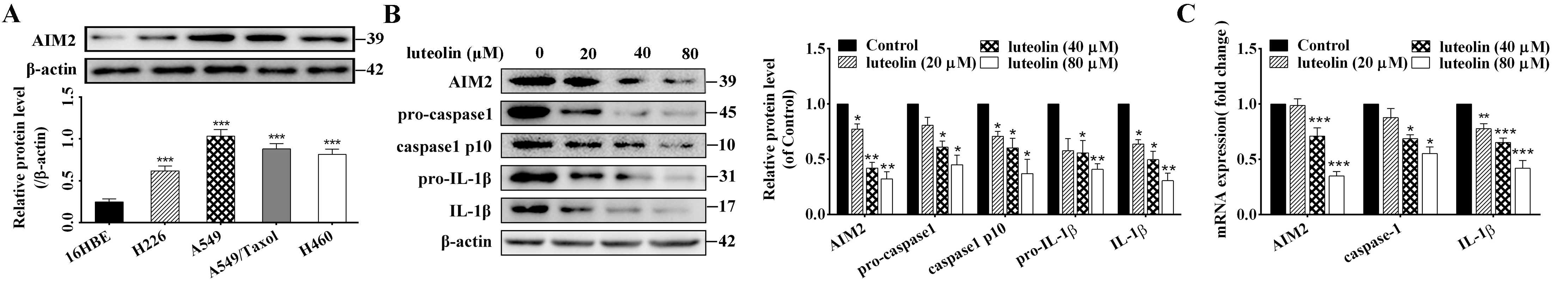

Supplement: Supplementary file 2 — Luteolin inhibited the activation of AIM2 inflammasome in H226 cells [file 41419_2019_1447_MOESM2_ESM.tif]

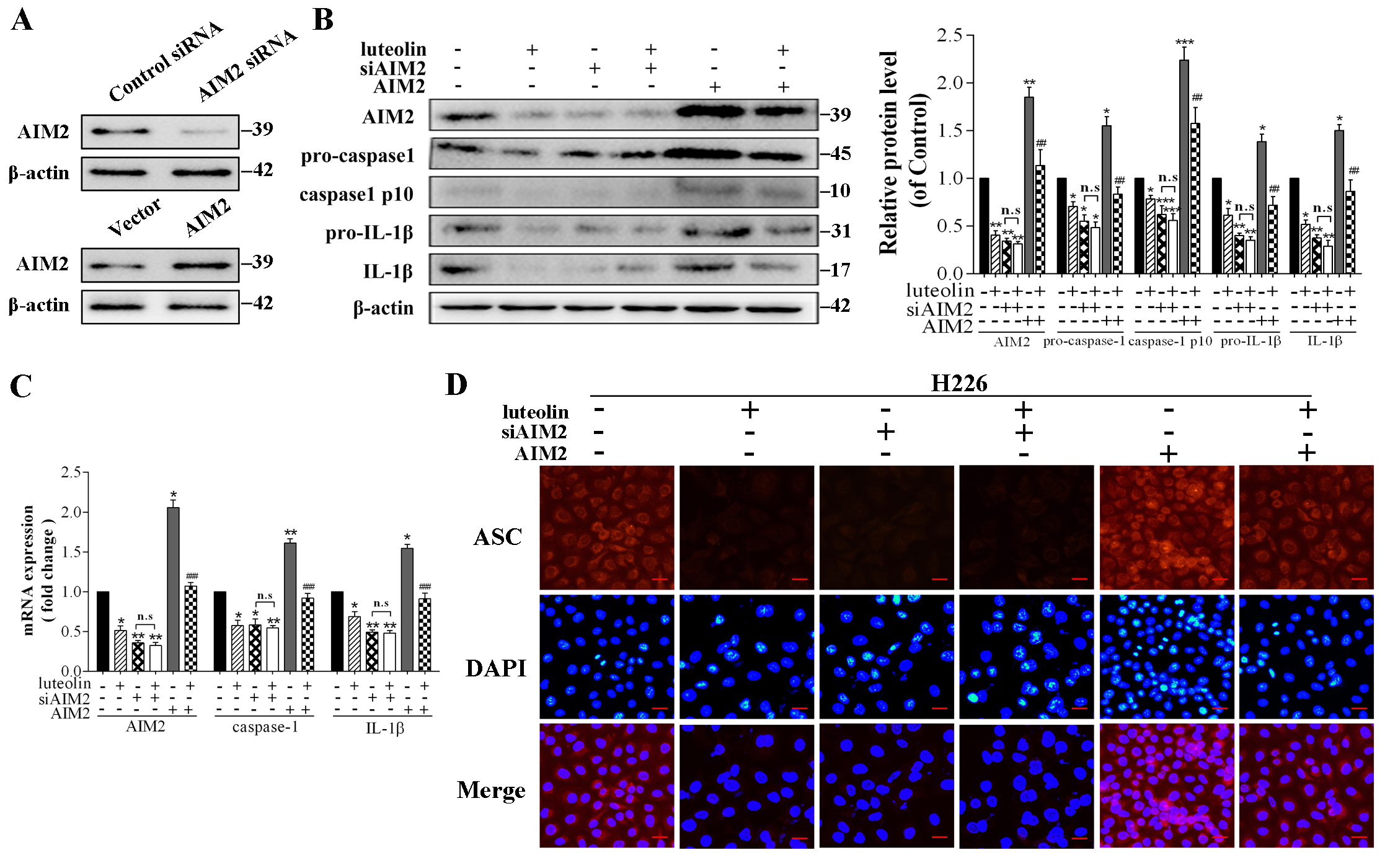

Supplement: Supplementary file 3 — Luteolin suppressed AIM2 inflammasome through downregulating the expression of AIM2 in H226 cells [file 41419_2019_1447_MOESM3_ESM.tif]

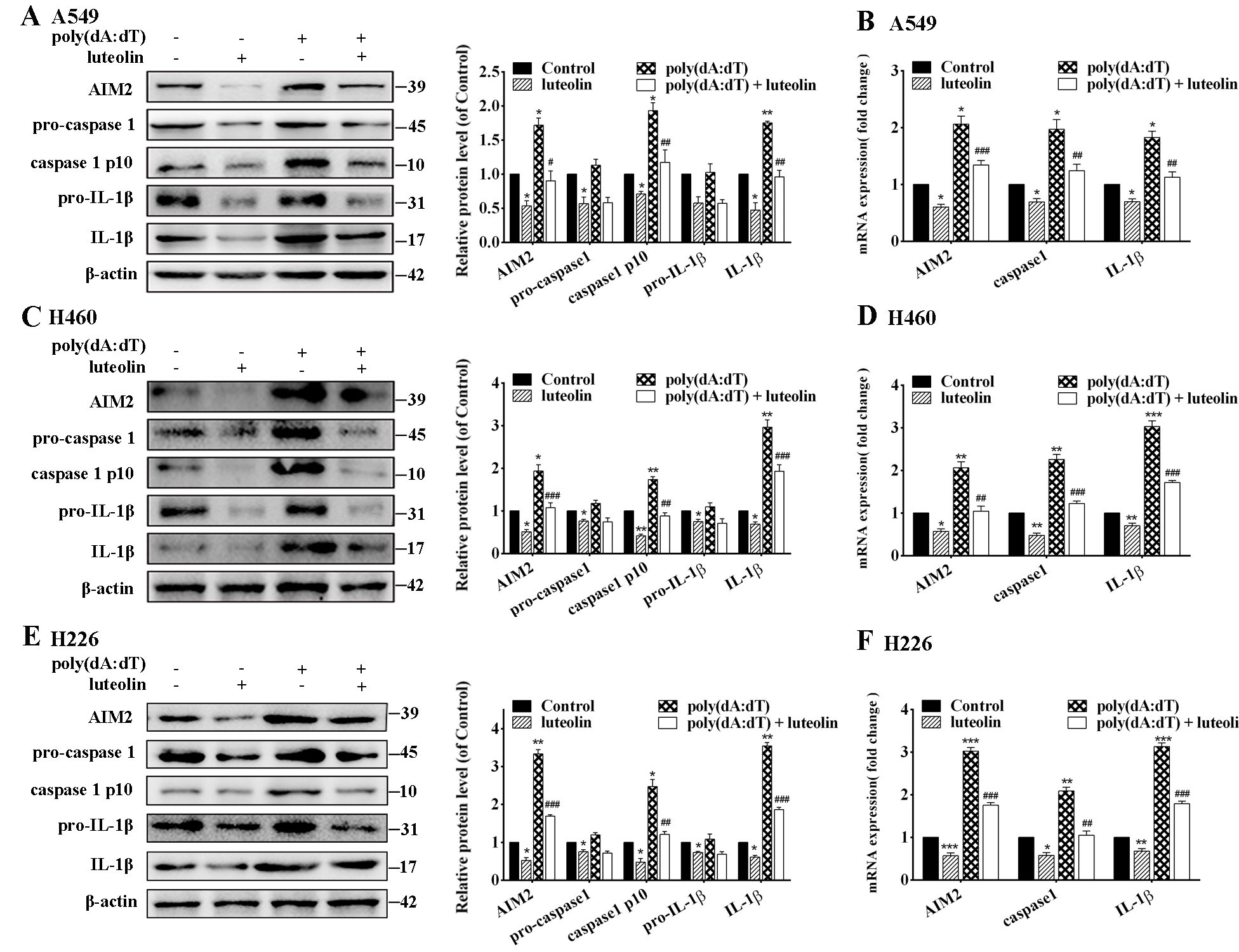

Supplement: Supplementary file 4 — Luteolin reduced poly(dA:dT)-induced caspase-1 activation and IL-1β maturation [file 41419_2019_1447_MOESM4_ESM.tif]

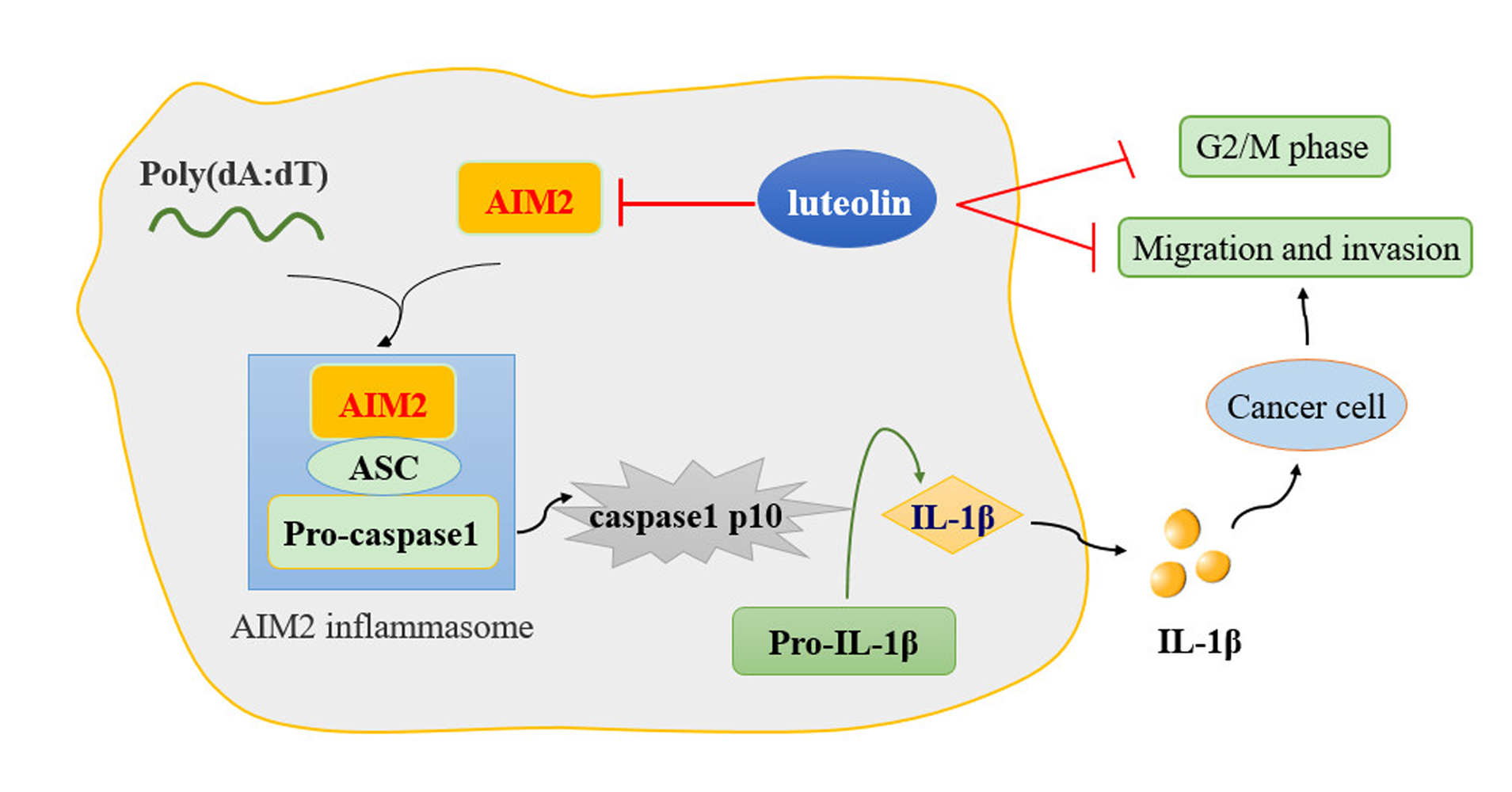

Supplement: Supplementary file 5 — Proposed mechanisms illustrating the anti-tumor effects of luteolin on NSCLC cells [file 41419_2019_1447_MOESM5_ESM.tif]
