# Supplementary material for: Decrease of AIM2 mediated by luteolin contributes to non-small cell lung cancer treatment
Source: Cell Death Dis. 2019 Mar 4;10(3):218. doi: 10.1038/s41419-019-1447-y (PMC6399355; doi:10.1038/s41419-019-1447-y)
Supplement: Supplementary file 6 — Supplemental figure legends [file 41419_2019_1447_MOESM6_ESM.docx]

**Supplementary Figure S1.** **The effects of luteolin in 16HBE, H226 and A549/Taxol cells.** (A, B and C) MTT assay for cell growth of 16HBE, H226 and A549/Taxol cells after treatment with different concentrations of luteolin. (D) H226 cells were treated with luteolin at different concentrations for indicated time. Cell morphology was captured by Nikon microscope (scale bar = 100 μm). (E) Representative images of the colony formation assay for H226 cells. **P* < 0.05, ** *P*< 0.01, ****P* < 0.001 *vs* control.

**Supplementary Figure S2. Luteolin inhibited the activation of AIM2 inflammasome in H226 cells.** (A) Western blot assay for protein expression of AIM2 in 16HBE, H226, A549, A549/Taxol and H460 cells. (B) The expressions of AIM2, pro-caspase1, caspase-1 p10, pro-IL-1β and IL-1β in H226 cells were determined by western blot. (C) RT-qPCR were performed to detect the mRNA levels of AIM2, caspase-1 and IL-1β in H226 cells. **P* < 0.05, ** *P*< 0.01, ****P* < 0.001 *vs* control.

**Supplementary Figure S3. Luteolin suppressed AIM2 inflammasome through downregulating the expression of AIM2 in H226 cells.** (A) Western blot analysis of AIM2 expression in H226 cells transfected with siAIM2 or AIM2 overexpression plasmid. (B and C) H226 cells were pretreated with siAIM2 or AIM2 overexpression plasmid before treating with or without luteolin (40 μM) for indicated time. The expressions of AIM2, pro-caspase1, caspase-1 p10, pro-IL-1β and IL-1β were determined by western blot. RT-qPCR were performed to detect the mRNA levels of AIM2, caspase-1 and IL-1β. (D) Cells were pretreated with siAIM2 or AIM2 overexpression plasmid before treating with or without luteolin for indicated time to detect the localization of ASC by immunofluorescence (scale bar = 25 μm). **P* < 0.05, ** *P*< 0.01, ****P* < 0.001 *vs* control, ^#^ *P* < 0.05, ^##^ *P* < 0.01, ^###^ *P* < 0.001 *vs* luteolin (40 μM), NS means no significant difference.

**Supplementary Figure S4. Luteolin reduced poly(dA:dT)-induced caspase-1 activation and IL-1β maturation.** Cells were stimulated with or without poly(dA:dT), followed by treatment with or without luteolin (40 μM) for indicated times. (A, C and E) Western blot was performed to detect the expressions of AIM2, pro-caspase1, caspase-1 p10, pro-IL-1β and IL-1β in A549, H460 and H226 cells, respectively. (B, D and F) RT-qPCR was performed to detect the mRNA levels of AIM2, caspase-1 and IL-1β in A549, H460 and H226 cells, respectively. **P* < 0.05, ***P* < 0.01, ****P* < 0.001 *vs* control, ^#^ *P* < 0.05, ^##^ *P* < 0.01, ^###^ *P* < 0.001 *vs* luteolin (40 μM).

**Supplementary Figure S5. Proposed mechanisms illustrating the anti-tumor effects of luteolin on NSCLC cells.**
